# Functional Homology for Antibody-Dependent Phagocytosis Across Humans and Rhesus Macaques

**DOI:** 10.3389/fimmu.2021.678511

**Published:** 2021-05-20

**Authors:** Justin Pollara, Matthew Zirui Tay, R. Whitney Edwards, Derrick Goodman, Andrew R. Crowley, Robert J. Edwards, David Easterhoff, Haleigh E. Conley, Taylor Hoxie, Thaddeus Gurley, Caroline Jones, Emily Machiele, Marina Tuyishime, Elizabeth Donahue, Shalini Jha, Rachel L. Spreng, Thomas J. Hope, Kevin Wiehe, Max M. He, M. Anthony Moody, Kevin O. Saunders, Margaret E. Ackerman, Guido Ferrari, Georgia D. Tomaras

**Affiliations:** ^1^ Department of Surgery, Duke University School of Medicine, Durham, NC, United States; ^2^ Human Vaccine Institute, Duke University School of Medicine, Durham, NC, United States; ^3^ Thayer School of Engineering, Dartmouth College, Hanover, NH, United States; ^4^ Department of Cell and Developmental Biology, Feinberg School of Medicine, Northwestern University, Chicago, IL, United States

**Keywords:** phagocytosis, Fc Receptor, rhesus macaques, antibody function, IgG3

## Abstract

Analyses of human clinical HIV-1 vaccine trials and preclinical vaccine studies performed in rhesus macaque (RM) models have identified associations between non-neutralizing Fc Receptor (FcR)-dependent antibody effector functions and reduced risk of infection. Specifically, antibody-dependent phagocytosis (ADP) has emerged as a common correlate of reduced infection risk in multiple RM studies and the human HVTN505 trial. This recurrent finding suggests that antibody responses with the capability to mediate ADP are most likely a desirable component of vaccine responses aimed at protecting against HIV-1 acquisition. As use of RM models is essential for development of the next generation of candidate HIV-1 vaccines, there is a need to determine how effectively ADP activity observed in RMs translates to activity in humans. In this study we compared ADP activity of human and RM monocytes and polymorphonuclear leukocytes (PMN) to bridge this gap in knowledge. We observed considerable variability in the magnitude of monocyte and PMN ADP activity across individual humans and RM that was not dependent on FcR alleles, and only modestly impacted by cell-surface levels of FcRs. Importantly, we found that for both human and RM phagocytes, ADP activity of antibodies targeting the CD4 binding site was greatest when mediated by human IgG3, followed by RM and human IgG1. These results demonstrate that there is functional homology between antibody and FcRs from these two species for ADP. We also used novel RM IgG1 monoclonal antibodies engineered with elongated hinge regions to show that hinge elongation augments RM ADP activity. The RM IgGs with engineered hinge regions can achieve ADP activity comparable to that observed with human IgG3. These novel modified antibodies will have utility in passive immunization studies aimed at defining the role of IgG3 and ADP in protection from virus challenge or control of disease in RM models. Our results contribute to a better translation of human and macaque antibody and FcR biology, and may help to improve testing accuracy and evaluations of future active and passive prevention strategies.

## Introduction

Fc receptors (FcRs) are cell surface proteins that interact with the Fc domains of antibodies to mediate cell signaling and effector functions (1, 2). Results of immune correlates analyses for the RV144 and HVTN505 clinical trials and preclinical vaccine studies conducted in rhesus macaques (RMs) have identified associations between non-neutralizing FcR-dependent antiviral activities of antibodies and reduced risk of HIV/SHIV infection or control of viremia (3–15). Among the myriad of potential FcR-dependent effector functions, antibody-dependent phagocytosis (ADP) of HIV-1/SIV envelope (Env) protein or virions has emerged as a unifying correlate of reduced infection risk in several vaccine studies conducted in RMs (3, 4, 6, 8), and in *post-hoc* analysis of the human HVTN505 HIV-1 vaccine efficacy trial (16). Despite this commonality, it remains unknown how effectively ADP activity observed in the RM model can predict that in humans.

Outcomes of a signaling event between antibody–antigen immune complexes and FcR are impacted by genetically-determined characteristics of the antibody including isotype, subclass, and allele, as well as characteristics of the FcR such as type, expression level, allele, and isoform (17). In addition, the composition of glycans on the antibody Fc and FcR also impacts interactions between immune complexes and FcR, providing a non-genetic level of control for signaling (18, 19). The downstream cellular response is largely dependent on the type of cell interacting with the immune complex. FcRs are variably expressed on the surface of different types of leukocytes and are therefore among the many clusters of differentiation that are used to define specific types of immune cells. Leukocytes can simultaneously express both activating and inhibitory FcRs, and the balance of these divergent signal pathways is critical to regulation of each potential effector response (1, 20). Although closely related, there are many differences in Fc/FcR biology between humans and RM including antibody subclass diversity (17), antibody structures (21–23), FcR genetic diversity (21, 22), Fc–FcR biophysical interactions (24–26), and cellular expression of FcR (23, 27–29). Thus, there is a need for direct comparisons of ADP between humans and RM to improve our understanding of how to most effectively use and translate results from this important animal model to future human interventions.

In this study, we compared the ADP activity of phagocytes from humans and RM using a panel of monoclonal antibodies (mAbs) specific for the CD4-binding site (CD4bs) of the HIV-1 Env protein that were recombinantly produced as different isotypes and subclasses. Peripheral blood monocytes and polymorphonuclear leukocytes (PMN) were used as sources of phagocytes. Our data demonstrated that, for both human and RM phagocytes, *in vitro* ADP activity was greatest when mediated by human IgG3, followed by RM IgG1, and then human IgG1. ADP activity among the tested antibody isotypes and subclasses was lowest, and often not detectable, when mediated by human IgA mAbs. We also observed considerable variability in the magnitude of monocyte and PMN ADP activity across individual humans and RMs. We found that these differences in human and RM ADP activity were not dependent on FcR alleles, and only modestly impacted by cell-surface levels of FcRs. Notably, we also describe production and characterization of novel RM IgG1 engineered with elongated hinge regions, and demonstrate that elongation of the RM IgG1 hinge to lengths similar to those of human IgG3 improves ADP activity mediated by RM monocytes and PMN. Our data contributes to the creation of a roadmap for effective translation of Fc–FcR biology across species that is needed to effectively evaluate non-neutralizing antibody effector functions in RMs and to make accurate predictions of human outcomes from preclinical studies. Moreover, the novel hinge variant antibodies we describe are expected to be useful surrogates for evaluating the protective or therapeutic potential of IgG3 antibodies of clinical interest in the preclinical RM model.

## Materials and Methods

### Study Samples and Reagents

#### Peripheral Blood Collection

Peripheral blood was collected by venipuncture, under sedation, from healthy adult RM at the Duke University School of Medicine, prior to allocation into any interventional research study in accordance with a protocol approved by the Duke University Institutional Animal Care and Use Committee. Human peripheral blood samples were collected by venipuncture of healthy consenting adult volunteers in accordance with a protocol approved by the Duke Health Institutional Review Board. Human and RM blood was collected into Acid Citrate Dextrose Vacutainer tubes (BD Biosciences, San Jose, CA) and processed within 4 hours of collection.

#### Isolation of Monocytes From Human and RM Peripheral Blood

Monocytes were isolated from the peripheral blood mononuclear cell (PBMC) fraction of fresh peripheral blood. PBMC were isolated using density gradient centrifugation with Ficoll-Paque PLUS (GE Healthcare Life Sciences, Pittsburg, PA). Monocytes were enriched from RM and human PBMC by positive selection using species-specific CD14 magnetic microbeads (Miltenyi Biotec) according to the manufacturer’s recommended protocol.

#### Isolation of PMN From Human and RM Peripheral Blood

Red blood cells were lysed from fresh whole blood using Red Blood Cell Lysis Solution (Miltenyi Biotec, Bergisch Gladbach, Germany). A PMN-enriched cell population was isolated from red blood cell-depleted blood by positive selection of granulocytes using anti-CD66abce magnetic microbeads (Miltenyi Biotec) (30) according the manufacturer’s recommended protocol.

#### Human and Rhesus-ized Monoclonal Antibody (mAb) Production

Human mAbs specific for the CD4bs site of HIV-1 Env, CH31 (31), and VRC01 (32), were expressed as human IgG1, IgG3 (IGHG3*01 allele), IgA1, IgA2, and RM IgG1 by transient cotransfection of heavy and light chain plasmids into Expi293-F cells with Expifectamine (Thermo Fisher Scientific, Waltham, MA). Secreted antibody was purified from cell culture supernatants by protein A or G resin columns as previously described (33, 34). The influenza specific CH65 mAb (35) produced as human IgG1, IgG3, IgA1, and IgA2 were used as negative controls for experiments with human antibodies. For experiments with RM IgG1, the V1V2-specific mAb HG107 (11) was produced as RM IgG1 (described below) and used as a negative control as this antibody does not bind to V1V2 of the subtype B Bal Env. The quality of all recombinant mAbs was evaluated using reduced and non-reduced SDS-PAGE followed by Coomassie stain or Western blot.

Rhesus-ization of antibodies was performed as previously described with some modifications (34). To generate VRC01 with a RM constant region, the human gamma constant region and kappa light chain constant region were exchanged for rhesus IgG1 constant regions (34). To produce rhesus-ized CH65 antibody the unmutated common ancestor antibody that gave rise to the human antibody lineage was first inferred using Cloanalyst (36). Using the inferred human germline antibody sequence, the macaque gene segments with the highest sequence homology were identified by searching the macaque library in Cloanalyst. All three affinity-matured complementarity-determining regions (CDRs) of the CH65 human heavy chain variable region (VH) or light chain variable region (VL) were grafted into the inferred macaque germline genes (**Supplemental Figure S1**). These variable regions were then attached to RM constant regions to form full-length immunoglobulin chains. The sequences were codon optimized using GeneOptimizer (GeneArt), synthesized *de novo* (GenScript, Piscataway, NJ), and expressed as described above.

RM IgG1 variants with extended hinges were designed with sequential repeats (0X, 1X, 2X, 3X and 4X) of the 18 amino acid (AA) RM hinge region between the CH1 and CH2 domains of RM IgG1. Antibody genes were synthesized (GenScript) similar to that previously described for human IgG1 and IgG3 (37). These antibodies were expressed and purified as described above.

### Laboratory Methods

#### Monocyte Antibody-Dependent Phagocytosis (ADP) Assay

Monocyte ADP of fluorescently-labeled HIV-1 BaL virions (38–40) was measured as previously described (41). Briefly, 10 µL of fluorescent RFP-labeled HIV-1 BaL virions were mixed with 10 µL of recombinant human and RM monoclonal antibodies (mAbs) at a final concentration of 25 µg/mL for 2 hours at 37°C in round-bottom 96 well plates (Corning Life Sciences, Durham, NC) to permit formation of immune complexes. Monocytes isolated from human or RM peripheral blood were counted using a Muse Cell Analyzer (Milipore Sigma, Burlington, MA), and added to the immune-complex containing wells at 60,000 viable cells per well in 20 µL RPMI-1640 media supplemented with 10% FBS. Plates were sealed, shaken at 750 RPM, and centrifuged for 1 hour at 1200 x g in a 4°C centrifuge. After centrifugation, the plate seal was removed and the plate was incubated for 1 hour at 37°C in a 5% CO_2_ atmosphere before being washed in 1% FBS PBS (wash buffer, WB), and fixed with a 4% formaldehyde PBS solution. Data acquisition and data analysis were performed as previously described (41), using a BD LSRFortessa flow cytometer and FlowJo Software (v9.9.6, BD Biosciences). The cytometer has been optimized and maintained using quality control procedures described by Perfetto and colleagues (42). ADP activity is presented as the phagocytosis score, calculated by percentage of cells positive × median fluorescence intensity (MFI), normalized by division with the corresponding result for the no-antibody control. An example of ADP assay data and calculation of scores is included as **Supplemental Figure S2**. Assays were performed in duplicate for each animal.

#### PMN Antibody-Dependent Phagocytosis (ADP) Assay

For PMN ADP of fluorescently-labeled HIV-1 subtype B BaL virions, immune complexes were formed as described as above. PMN isolated from human or RM peripheral blood were added to immune-complexes in 96-well plates at 60,000 viable cells per well in 200 µL RPMI-1640 media supplemented with 10% FBS and 1% HEPES. The plates were incubated at room temperature for 15 minutes, then centrifuged at room temperature for 1 minute at 300 x g, followed by a 1 hour incubation at 37°C in a 5% CO_2_ atmosphere. Plates were then washed, fixed, acquired, and analyzed as described previously (41), and above.

#### Binding and Affinity to FcRs

The affinity of Fc*γ* receptors (FcγR) for our IgG mAbs was measured as previously described (25). Briefly, a Continuous Flow Microspotter (CFM, Carterra, Salt Lake City, UT) and carbodiimide chemistry was used to immobilize the antibodies on a medium density carboxymethyldextran sensorchip (Xantec Bioanalytics, Düsseldorf, DEU). An 8-point series of 1:3 dilution of the receptor beginning at 20 µM was then flowed over the sensor surface and the association and dissociation were measured for 5 minutes each using an imaging-based surface plasmon resonance (SPRi) instrument (MX96, IBIS Technologies, Pantheon 5, NLD). The results were analyzed in Scrubber 2 (BioLogic Software, Campbell Australia) using a steady state model to determine the equilibrium dissociation constants.

#### Quantification of FcRs on the Surface of Human and RM Monocytes and PMN

Frequencies of FcR-bearing effector cells and quantification of the amount of FcR on cell surfaces was determined by immunofluorescence staining of fresh whole blood. Briefly, 100 µL of human or RM peripheral blood was incubated at room temperature, protected from light, for 25 minutes with the following combination of fluorescently conjugated monoclonal antibodies: PE-CF594-CD3 (clone SP34-2, BD Biosciences); PE-CF594-CD20 (clone 2H7, BD Biosciences); APC-Cy7-CD14 (clone MϕP9, BD Biosciences); PacificBlue-CD16 (clone 3G8, BD Biosciences); APC-CD32 (clone FL18.26, BD Biosciences); Alexa Fluor 700-CD64 (clone 10.1, BD Biosciences); PE-CD89 (clone A59, BD Biosciences); PerCP-Vio700-CD66 (clone TET2, Miltenyi Biotec); PE-Cy7-CD11b (clone ICRF44, Biolegend); FITC-CD62L (clone SK11, BD Biosciences); and PE-Cy5-CD49d (clone 9F10, BD Biosciences). After incubation, 2 mL of BD Pharm Lyse solution (BD Biosciences) was added to each tube, and incubated for 15 minutes at room temperature to lyse red blood cells. Leukocytes were subsequently pelleted by room temperature centrifugation for 5 minutes at 500 x g. The cell pellet was then washed with buffered saline and stained with a viability marker (Fixable Aqua Dead Cell Stain Kit, Thermo Fisher Scientific, Waltham, MA) for 20 minutes at room temperature. After two washes with 2 mL WB the cells were fixed in 1% paraformaldehyde buffer prior to data acquisition using our BD LSRFortessa flow cytometer. Quantum™ Simply Cellular^®^ beads (Bangs Laboratories, Inc., Fishers, Indiana) were used to determine the antibody binding capacity (ABC) of FcR on the surface of cells according the manufacturers recommended procedure. Data analyses were performed using FlowJo software (v9.9.6, BD Biosciences).

#### FcR Sequence Analysis

FcR sequence analysis was performed using long-read RNA sequencing. RNA and genomic DNA were isolated from human and RM PBMC samples using the AllPrep DNA/RNA isolation kit (Qiagen, Germantown, MD). RNA was reverse transcribed using the Qiagen QuantiTect Reverse Transcription Kit (Qiagen) and Fc*γ*R gene-specific primers designed with PacBio barcodes (Pacific Biosciences, Menlo Park, CA). PCR products were purified using ZR-96 DNA clean and Concentrator™-5 (Zymo Research) following manufacturer’s protocol. PacBio SMRTbell library preparation was performed in accordance with manufacturer’s recommendations (Pacific Biosciences) and equal concentrations of ~30 amplicons were pooled and loaded onto a single SMRT cell as determined by Qubit quantification (ThermoFisher Scientific). Sequencing was performed on a PacBio Sequel II instrument using 2.1 or 3.0 chemistry (Pacific Biosciences). Datasets were loaded into PacBio SMRT Link 7.0.1 software package for demultiplexing of subreads and generating circular consensus sequences (CCS). And quality control was assessed by analyzing productivity and sequence read length before and after trimming. Data analysis was conducted using a pipeline similar to that previously described (43). Each unique CCS was aligned to GenBank deposited reference sequences using a long-read sequence alignment tool (44) and variants were identified using the GATK Haplotype Caller (45), and annotated with ANNOVAR (46).

#### Negative Stain Electron Microscopy

Antibody samples were diluted to 20 µg/ml with buffer containing 150 mM NaCl, 0.014 g/dL ruthenium red, and 20 mM HEPES buffer, pH 7.4, and incubated at room temperature for at least one hour. A 5-µl drop of diluted antibodies were applied to a glow-discharged carbon film covered 300 mesh copper EM grid and incubated 10 seconds, blotted with filter paper, rinsed with a 5 µl drop of buffer containing 7.5 mM NaCl and 1 mM HEPES, pH 7.4, for ~7 seconds, blotted and then stained with 0.6% uranyl formate for 1 minute. Excess stain was blotted and the grids were allowed to air dry. Grids were imaged in a Philips EM420 electron microscopy at 120 kV and 82,000x, and 60-80 images per sample with ~1000 particles per image were captured with a 2k x 2k CCD camera at a pixel size of 4.02 Å/pixel. Images were imported into the Relion software package and 2D class averages were calculated by standard methods (47).

#### SHIV-Infection of A66 Cells

SHIV.Bal(P4) virus stocks (48) grown in human PBMCs were titrated to determine the input required for optimal viral gene expression within 72 h post-infection of A66 cells as measured by intracellular p27 expression (WNPRC Immunology Services). A66 cells (provided by Dr. James Hoxie, University of Pennsylvania, Philadelphia, PA) are SupT1 cells (non-BC7 variant (49)) that have been stably transfected to express both rhesus CD4 and rhesus CCR5 receptors after knockout of endogenous human CXCR4 and CD4 (50). SHIV.BaL(P4) was used to infect 1 × 10^6^ A66 cells by incubation with 24 ng/mL of p27 for 4 hours at 37°C and 5% CO_2_ in the presence of DEAE-Dextran (10 μg/mL, Sigma Aldrich). The cells were subsequently resuspended at 0.33 × 10^6^/mL and cultured for 3 days in complete medium containing 10 μg/mL DEAE-Dextran. On assay day, infection was monitored by measuring the frequency of cells expressing intracellular p27. The assays performed using the SHIV-infected target cells were considered reliable if the percentage of viable p27+ target cells on assay day was ≥10%. Assay data generated using infected cells was normalized to the frequency of live target cells positive for intracellular p27.

#### Infected Cell Antibody Binding Assay (ICABA)

ICABA was used to evaluate the ability of RM IgG hinge variant mAbs to bind Env on the surface of SHIV-infected cells. SHIV-infected A66 cells were obtained as described above. Cells incubated in the absence of virus (mock infected) were used as a negative control. Infected and mock infected cells were washed in PBS, dispensed into 96-well V-bottom plates at 2 x 10^5^ cells/well and incubated with 1 μg/mL of indicated mAbs for 2 hours at 37°C. After two washes with 250 μL/well WB, the cells were stained with vital dye (Live/Dead Fixable Aqua Dead Cell Stain, Invitrogen) to exclude nonviable cells from subsequent analysis. Cells were washed with WB and stained with anti-CD4-PerCP-Cy5.5 (clone Leu-3; BD Biosciences) to a final dilution of 1:20 in the dark for 20 min at room temperature (RT). Cells were then washed again, and permeabilized using Cytofix/Cytoperm (BD Biosciences). Anti-p27 antibody (WNPRC Immunology Services, 1:500 dilution in 1x Cytoperm Solution, BD Biosciences) and a secondary PE-conjugated antibody (goat anti-human Ig Fc-PE, eBioscience, San Diego, CA., final dilution of 1:400) were added to each well and incubated in the dark for 25 min at 4°C. Cells were washed three times with Cytoperm wash solution and resuspended in PBS-1% paraformaldehyde. The samples were acquired within 24 hours using a BD Fortessa cytometer. A minimum of 50,000 total events was acquired for each analysis. Gates were set to include singlet and live events. Data analysis was performed using FlowJo 9.6.6 software (BD Biosciences). Final data represents the PE MFI of binding of IgG mAbs to HIV Env, after normalization by subtraction of the PE MFI observed for cells stained with the secondary antibody alone. Assays were repeated twice and the average of the results is shown.

#### Antibody-Dependent Cell-Mediated Cytotoxicity (ADCC)

We used an infected cell elimination assay to measure ADCC activity of RM IgG hinge variant mAbs. SHIV-infected or mock-infected A66 cells were used as targets and NHP PBMCs rested overnight in R10 were used as a source of effector cells. On assay day, infected and uninfected target cells were washed in R10 and labelled with a fluorescent target-cell marker (TFL4; OncoImmunin) and a viability marker (NFL1; OncoImmunin) for 15 min at 37°C, as specified by manufacturer. Cells were washed in R10 and adjusted to a concentration of 0.2 x 10^6^ cells/mL. PBMCs were then added to target cells at an effector/target ratio of 60:1 (12 x 10^6^ cells/mL). The target/effector cell suspension was plated in V-bottom 96-well plates and co-cultured with each mAb at the starting concentration of 50 μg/mL with subsequent three dilutions at 1:10. Co-cultures were incubated for 6 hours at 37°C in 5% CO_2_. After the incubation period, cells were washed and stained with anti-CD4-PerCP-Cy5.5 (BD Biosciences, clone Leu-3) at a final dilution of 1:20 in the dark for 20 min at RT. After washing with WB, cells were resuspended in 100 μL/well Cytofix/Cytoperm (BD Biosciences), incubated in the dark for 20 min at 4°C, washed in 1x Cytoperm wash solution (BD Biosciences) and co-incubated with anti-p27 antibody (WNPRC Immunology Services) to a final dilution of 1:500, and incubated in the dark for 25 min at 4°C. Three washes were performed with Cytoperm wash solution before resuspending the cells in 125 μL PBS-1% paraformaldehyde for acquisition. The samples were acquired within 24 h using a BD Fortessa cytometer. The appropriate compensation beads were used to compensate the spill over signal for the four fluorophores. Data analysis was performed using FlowJo 9.6.6 software (TreeStar). Mock-infected cells were used to appropriately position live cell p27+/- gates. Specific killing was determined by the reduction in % of p27+ cells in the presence of mAbs after taking into consideration non-specific killing according to the following formula: percent specific killing = [(Frequency of p27 positive cells in wells containing targets and effectors alone − Frequency of p27 positive cells in wells containing targets and effectors with antibodies)/Frequency of p27 positive cells in wells containing targets and effectors alone] ×100. CH65 (anti-influenza mAb) produced as human and RM IgG1 were used as negative controls. Assays were repeated twice and final data represents the mean and range of results.

### Statistical Analysis

All statistical analysis was performed using SAS software (version 9.4; SAS Institute Inc., Cary, N.C.). Kruskal Wallis tests were used to compare response magnitudes between groups. In order to assess if two groups had different responses pairwise comparisons between groups were conducted using Wilcoxon rank sum tests. A p-value of less than 0.05 was considered to be statistically significant. Spearman’s rank correlation coefficient was used to assess correlation between ADP activities of different antibody isotypes and the amount of cell-surface FcR.

## Results

### HIV-1 Virion ADP Activity of Human and RM Monocytes and PMN From Peripheral Blood

**Figure 1:**
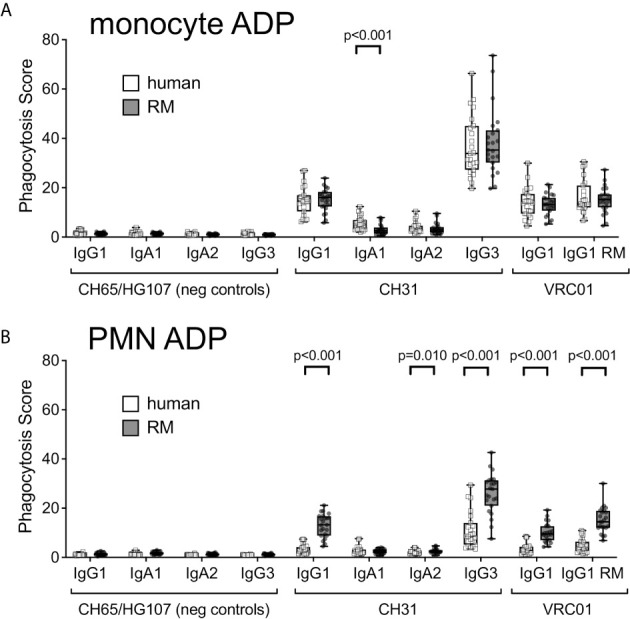
ADP of immune complexes formed with fluorescent HIV-1 BaL virions and human or RM monoclonal antibodies, as indicated, in assays performed using **(A)** monocytes, and **(B)** PMN, isolated from peripheral blood of 23 humans (white boxes, square symbols) or 23 RM (gray boxes, round symbols), as sources of phagocytes. Box plots represent the interquartile ranges, horizontal lines indicate the medians, and error bars represent the range.

Although associations between vaccine-elicited ADP responses and reduced infection risk have been observed in multiple preclinical vaccine studies conducted in RM (3, 4, 6, 8) and in the HVTN505 HIV-1 vaccine efficacy trial (16), it remains unknown how effectively ADP activity in the RM model can predict that of humans. To address this limitation, we compared the ADP activity of RM and human monocytes and PMNs using anti-HIV-1 CD4 binding site-specific mAbs CH31 and VRC01. Standard recombinant production techniques (33) were used to produce the mAbs as human IgG1, IgG3, IgA1, and IgA2. To test the function of the rhesus constant region, the human heavy and kappa light chain constant regions of VRC01 were exchanged for rhesus IgG1 constant regions (called RM VRC01 IgG1 hereafter) (34). We allowed the antibodies to interact with fluorescent HIV-1 BaL virions to form immune complexes, which were then incubated with monocytes or PMN isolated from peripheral blood of 23 RM or 23 healthy human donors. The anti-influenza mAb CH65 (35) and the HIV-1 V1V2 mAb HG107 (11) were used as negative controls as neither mAb was expected to specifically bind the HIV-1 subtype B BaL virions used in these experiments. ADP of fluorescent virions was detected by flow cytometry, and was reported as a score ratio (41). In virion ADP assays performed with peripheral blood monocytes (**Figure 1A**), we observed no significant differences (Wilcoxon p>0.05) in ADP activity of human and RM monocytes for phagocytosis mediated by any of the antibodies tested with the exception of CH31 IgA. CH31 IgA1 ADP with human monocytes was higher than that observed with RM monocytes (Wilcoxon p<0.001, **Figure 1A**). As shown in **Figure 1B**, we found that ADP activity of RM PMN was significantly higher (Wilcoxon p<0.001) than that observed for human PMN when mediated by CH31 and VRC01 IgG1, CH31 IgG3, and RM VRC01 IgG1. Despite these differences in the magnitudes of ADP activity, we observed a similar rank order of ADP activity for different antibody isotypes and subclasses, regardless of whether assays were performed with human or RM monocytes or PMN. For human and RM phagocytes, ADP activity was greatest when mediated by human CH31 IgG3, followed by RM VRC01 IgG1 and human CH31 or VRC01 IgG1 (Wilcoxon p<0.001 for human IgG3 versus RM or human IgG1); and was lowest when mediated by human CH31 IgA mAbs. These results demonstrate that although there are differences in the absolute magnitude of ADP activity when comparing human and RM phagocytes, there is functional homology across these two species for ADP activity irrespective of species mismatch between antibody and FcRs. Thus, we observed conservation of relative functional profiles between humans and RM for the types of antibodies tested.

### Correlation of Human and RM ADP Activity Across Antibody Isotypes and Subclasses

**Figure 2:**
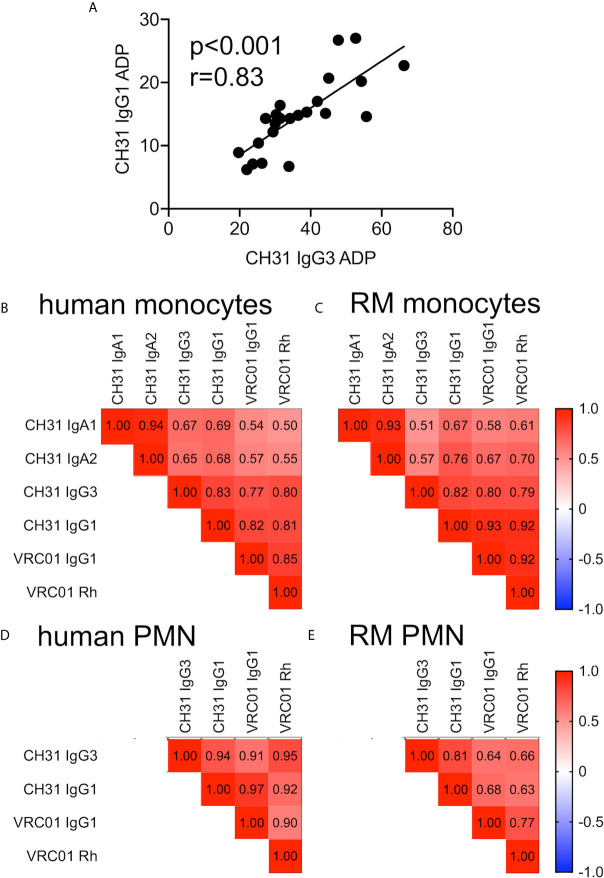
**(A)** Correlation of CH31 IgG1 and IgG3 ADP activities in assays performed with human monocytes (n=23). (**B–E**) Heatmaps of Spearman correlation coefficients (r values) for ADP activities of indicated antibody isotypes in assays performed with **(B)** human monocytes, **(C)** RM monocytes, **(D)** human PMN, and **(E)** RM PMN. IgA correlations were omitted for neutrophil ADP due to the majority of responses being similar to negative controls. The p values for all Spearman correlations are all ≤0.01.

As shown in **Figure 1**, we found that there was considerable variability in the magnitude of monocyte and PMN ADP activity across individual humans and RMs. We therefore used Spearman correlation testing to determine if ADP activity was correlated across antibody isotypes and subclasses to determine if phagocyte ADP activity was relatively high or low regardless of the composition of the immune complex involved. A representative scatter plot showing correlation of CH31 IgG1 and IgG3 ADP activities in assays performed with human monocytes is shown in **Figure 2A**. We found significant (Spearman p<0.05, r>0.5) positive correlations for ADP assays performed with human and RM monocytes (**Figures 2B, C**, respectively). Correlations were strongest when comparing subclasses within the IgG or IgA isotypes, and were weaker when comparing between IgG and IgA isotypes. We also observed significant (Spearman p<0.05, r>0.5) positive correlations for ADP assays performed with human and RM PMN (**Figures 2D, E**, respectively) across all ADP-mediating antibody isotypes and subclasses. Correlations were not evaluated for PMN ADP with CH31 IgA1 or IgA2 for humans (**Figure 2D**) and RM (**Figure 2E**) due to the majority of activities being similar to that observed with negative control IgA antibodies (**Figure 1B**). These analyses suggest that relative differences in ADP activity observed for individual humans and RM is related to the phagocytic propensity of the cells obtained from each blood donor, and is conserved across antibody isotype or subclass. Based on these results, we next sought to identify characteristics of RM and human peripheral blood phagocytes that contribute to differences in ADP activity across individuals.

### Impact of *FCGR2A* Allelic Diversity on ADP Activity of Human and RM Peripheral Blood Monocytes and PMN

**Figure 3:**
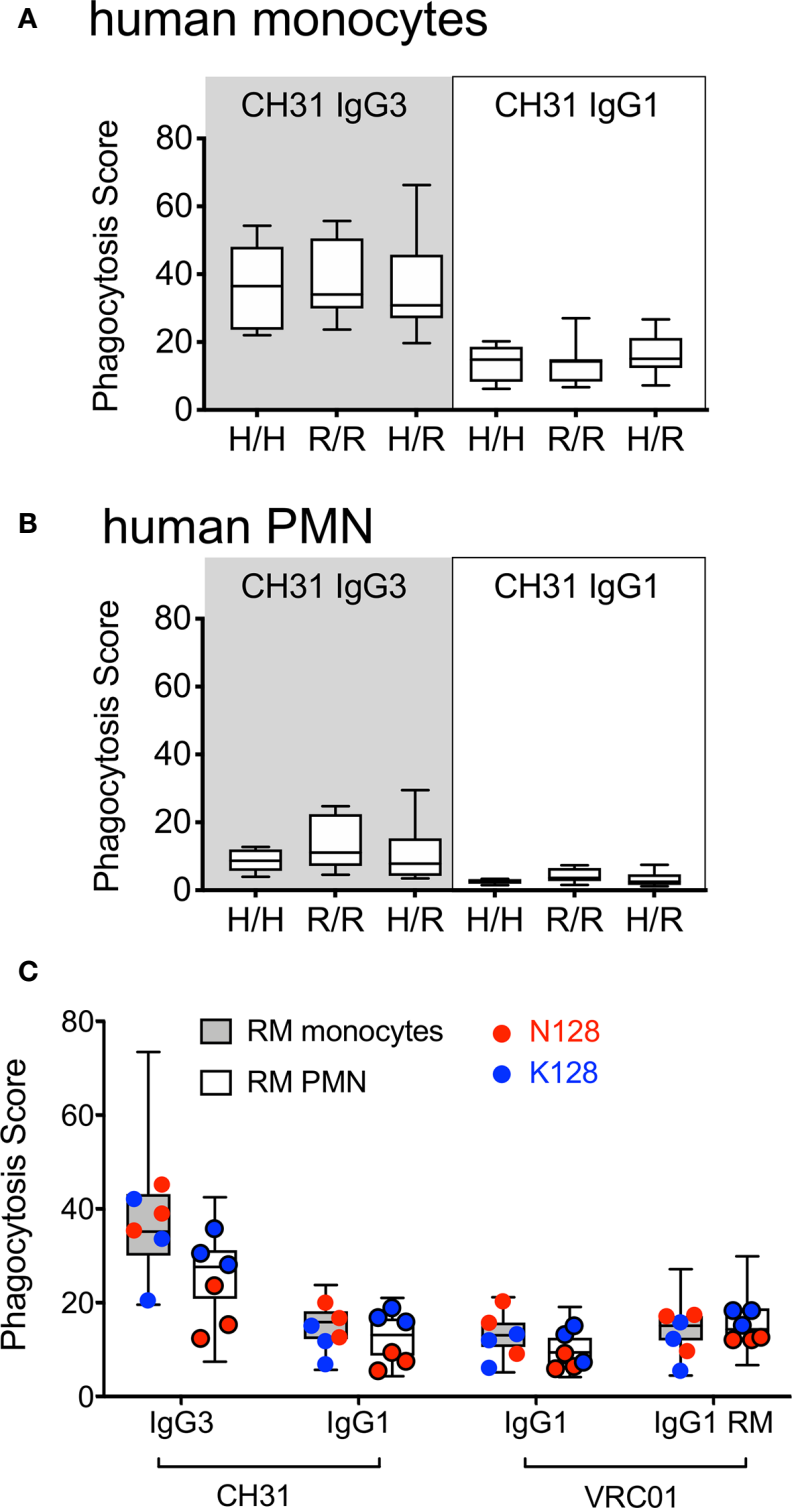
ADP activity of IgG3 and IgG1 mAbs in assays performed with **(A)** human monocytes or **(B)** human PMN when stratified by presence of Fc*γ*RIIa SNPs known to impact affinity for IgG. 5 donors were homozygous for H131 (H/H), 8 were homozygous for R131 (R/R) and 10 were heterozygous (H/R). **(C)** ADP activity observed for RM monocyte or PMN ADP activity when evaluated with respect to SNPs within the RM Fc*γ*RIIa IgG contact region. Genotype data was only available for 6 of the 23 animals tested, which are represented by symbols overlaying the total data (box-whisker plots). RM with identified SNPs within the IgG contact region are represented by red (N128) and blue (K128) circles as indicated. For all plots, boxes extend to the interquartile ranges, horizontal lines indicate the medians, and error bars represent the range.

Fc*γ*RIIa has been previously implicated in IgG-mediated ADP of antibody responses against HIV-1 (16, 51–54). Single nucleotide polymorphisms (SNPs) in the human *FCGR2A* gene (which encodes Fc*γ*RIIa) affecting an amino acid within the IgG contact region result in Fc*γ*RIIa allelic variants with lower (R131) or higher (H131) relative affinities for IgG (26). To determine if these SNPs contributed to the differences in human monocyte (**Figure 3A**) and PMN (**Figure 3B**) ADP activity observed across individual human donors, we stratified the results of testing with CH31 IgG3 and IgG1 mAbs by homozygosity for the high affinity H131 allele (H/H, n=5 donors), homozygosity for the low affinity R131 allele (R/R, n=8 donors), and heterozygosity (H/R, n=10 donors). We found no significant differences (Wilcoxon p>0.05) in IgG3 or IgG1 ADP activity when comparing between donor groups separated by *FCGR2A* alleles. Thus, these data indicate that SNPs that have an effect on the affinity of human Fc*γ*RIIa did not significantly impact the *in vitro* ADP activity of human monocytes and PMN in these experiments and therefore do not explain the differences we observed for ADP activity across individual humans. We next performed a similar analysis for RM phagocytes. Due to limitations in available cells for genotyping, we were only able to define the *FCGR2A* alleles of 6 of the 23 RM included in our cohort. One SNP within the IgG contact region was identified within these RM. Three animals had a potential N-linked glycosylation site at position 128 (N128), and the other three did not (K128). We found no consistent trend that would provide evidence of the amino acid composition at position 128 influencing stratification for IgG1 and IgG3 ADP activity of RM monocytes, but we did observe a trend for reduced ADP among RM PMN with N128 (**Figure 3C**).

### Impact of Cell-Surface Abundance of FcRs on ADP Activity of Human and RM Peripheral Blood Monocytes and PMN

**Figure 4:**
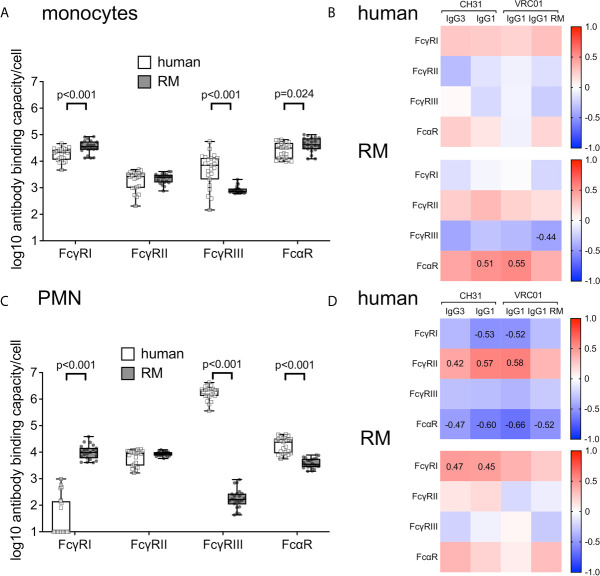
**(A)** Number of cell-surface FcR measured as antibody binding capacity on the surface of human (n=23, white boxes with square symbols) and RM monocytes (n=21, gray boxes with round symbols). **(B)** There was no significant correlation (Spearman correlation p values all > 0.05 and r values < 0.5) between amount of cell-surface FcR and ADP activity of indicated mAbs in assays performed with human monocytes (top panels) or RM monocytes (bottom panels). **(C)** Number of cell-surface FcR measured as antibody binding capacity on the surface of human (n=23, white boxes with square symbols) and RM (n=22, gray boxes with round symbols) PMN. **(D)** Spearman correlations between amount of cell-surface FcR and ADP activity of human (top panels) or RM PMN (bottom panels). Numbers on heatmaps indicate Spearman r values of significant correlations (p<0.05).

We next sought to determine if the amount of FcRs on the surface of human and RM monocytes and PMN correlate with ADP activity. We used flow cytometry phenotyping and fluorescent quantitation beads to detect and measure cell surface expression of FcRs. As shown in **Figure 4A**, human and RM monocytes express similar types of cell-surface FcRs; however, we observed significant differences in the levels of FcR expressed per cell. Levels of Fc*γ*RI and FcαR were lower on human monocytes when compared to RM monocytes (Wilcoxon p<0.001). Although human and RM blood contains similar frequencies of monocyte subsets when stratified by Fc*γ*RIII expression (55), we found higher levels of Fc*γ*RIII on human pan-monocytes compared to RM pan-monocytes (Wilcoxon p<0.001). Importantly, no differences were observed for cell-surface levels of Fc*γ*RII. We performed Spearman correlation analysis to determine if there was a relationship between the level of cell-surface FcRs and ADP activity. We found no-significant correlations (Spearman p>0.05 and r<0.5) between IgG3 and IgG1 ADP activity and cell-surface levels of FcR for human monocytes (**Figure 4B**, upper panel). Similarly, ADP activity of RM monocytes was not correlated with cell-surface levels of FcR with the exception of ADP by human IgG1 antibodies, which surprisingly were weakly correlated with amount of FcαR on the cell surface (**Figure 4B**, lower panel). However, we found that levels of RM Fc*γ*RII were positively correlated with levels of FcαR (Spearman r=0.60, p<0.005) on RM monocytes as shown in **Supplemental Figure S3**. Thus, one possible explanation for the association of an IgG functional response with FcαR abundance is that monocyte IgG ADP activity is, at least in part, influenced by the levels of cell-surface Fc*γ*RII, which has a direct relationship with FcαR for expression on the surface of RM monocytes.

We found significant differences between PMN cell-surface expression of FcRs when comparing cells from humans and RM (**Figure 4C**). Human peripheral blood PMN expressed significantly less cell-surface Fc*γ*RI (Wilcoxon p<0.001), but more Fc*γ*RIII (p<0.001) and FcαR (p<0.001) when compared to RM. The presence of cell-surface Fc*γ*RI and absence of Fc*γ*RIII (specifically the Fc*γ*RIIIb isoform) on RM PMN is consistent with prior observations (27–29). As described above for monocyte ADP, we performed Spearman correlation analysis to identify relationships between cell surface FcRs and PMN ADP activity. For human PMN, we found weak (Spearman r between 0.42 and 0.58) but significant (p<0.05) positive correlations between the amount of cell surface Fc*γ*RII and ADP activity of human IgG1 antibodies. We also found weak negative correlations (Spearman r < 0.6) between IgG1 ADP and cell-surface levels of Fc*γ*RI, and between IgG ADP activity and FcαR levels (Spearman r <0.7, **Figure 4D**, upper panel). Consistent with this observation, we found that human PMN cell-surface levels of Fc*γ*RII were negatively correlated with levels of Fc*γ*RI (Spearman r=-0.82) and levels of FcαR (Spearman r=-0.56, **Supplemental Figure S3**). These findings suggest that cell-surface levels of Fc*γ*RII impact the IgG1 ADP activity of human PMN, and that the abundance of this receptor is inversely associated with the abundance of FcαR and Fc*γ*RI —which is normally absent or present in only low levels on the surface of canonical resting human PMNs (56). Interestingly, these relationships were not identified for RM PMN ADP. In contrast, we found significant, but weak (r<0.5), correlations between the levels of Fc*γ*RI and on the surface of RM PMN and CH31 IgG1 and IgG3 ADP activity (**Figure 4D**, lower panel). Moreover, no significant relationships between the cell-surface levels of different cell surface FcR were observed for RM PMN (**Supplemental Figure S3**). These observations suggest that RM PMN ADP may be more dependent on signaling through Fc*γ*RI instead of Fc*γ*RII. In light of this possibility, we explored whether there was *FCGR1A* allele-dependent stratification for IgG1 and IgG3 ADP activity of RM PMN. However, within our RM cohort we identified only one SNP in *FCGR1A* that changes an amino acid within the IgG contact region, and this SNP (H158) was only represented in one animal. It is therefore unlikely that *FCGR1A* allelic variation underlies the differences in PMN ADP activity we observed across individual RM donors.

### Comparable Binding Affinities of Human IgG1 and IgG3 and RM IgG1 to Common Variants of Both Human and RM Fc*γ*Rs

**Figure 5:**
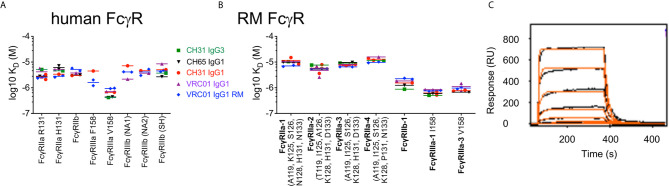
Human IgG3, and human and RM IgG1 mAbs have similar binding affinities for common variants of **(A)** human or **(B)** RM Fc*γ*Rs. Human and RM variants of Fc*γ*RIIa and Fc*γ*RIIIa are identified by SNPs in the IgG contact region, and human Fc*γ*RIIIb variants are identified by canonical haplotype name. Repeated symbols indicate results of technical replicates, and lines represent the means. Data for CH65 IgG1 binding to RM Fc*γ*RIIa-4 is not presented due to poor curve fit. **(C)** Example sensogram for CH31 IgG1 binding to human FcR*γ*IIa H131 at 200 nM print density. Data represents testing of an 8 point series of 3-fold dilutions of CH31 IgG starting at 20 µM. The orange curve represents the kinetic fit; however, the reported values in A and B were calculated using the steady state signal at the end of the association phase.

Although we identified differences in the magnitude of ADP across individuals, a consistent finding was that among all humans and RM in our study cohort, the highest ADP activity was observed for immune complexes formed with IgG3. In fact, phagocytosis scores for CH31 IgG3 were approximately double those observed for CH31 IgG1 in assays using human and RM monocytes or PMN (**Figure 1**). This superior ADP activity of IgG3 has previously been demonstrated for assays performed using human phagocyte cell lines and primary human phagocytes (41, 57, 58). Importantly, the unique elongation of the hinge region in human IgG3 subclass, and not affinity for Fc*γ*Rs, was shown to be responsible for the improved phagocytic potency of IgG3 when compared to IgG1 (37). We used multiplexed surface plasmon resonance (SPR) assays to evaluate the binding affinities of our panel of IgG1 and IgG3 antibodies to Fc*γ*Rs. We identified only minor differences in binding affinities of the antibodies used in our study to common variants of both human (**Figure 5A**) and RM (**Figure 5B**) Fc*γ*Rs. In **Figure 5**, human and RM variants of Fc*γ*RIIa and Fc*γ*RIIIa are identified by SNPs in the IgG contact region, and human Fc*γ*RIIIb variants are identified by canonical haplotype name (25, 59). An example sensogram for CH31 IgG1 binding to human FcR*γ*IIa H131 at 200 nM print density is shown in **Figure 5C** and complete SPR sensor data are included as **Supplemental Figures S4–S5**. These results suggest that as previously described for interactions with human phagocytes, the potent RM phagocyte ADP activity we observed for virion immune complexes formed with human IgG3 antibodies is likely dependent on IgG3 hinge length.

### Development of RM IgG1 With Elongated Hinge Regions

**Figure 6:**
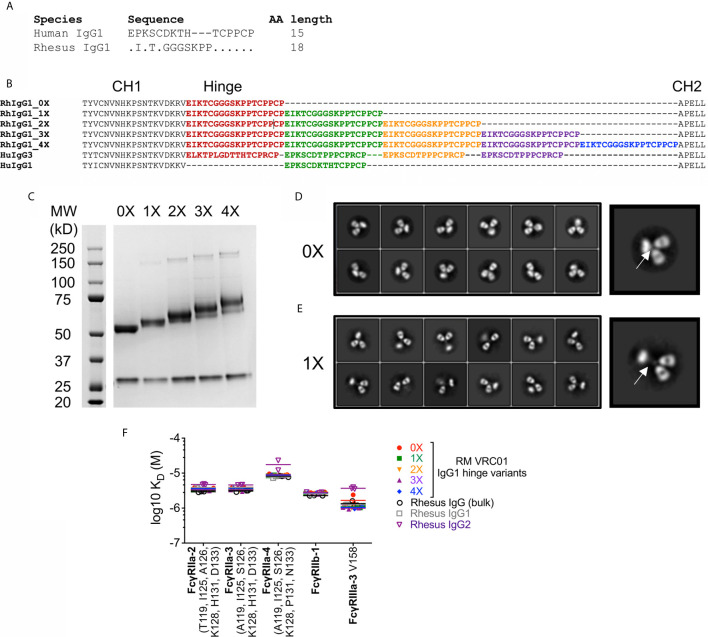
**(A)** Alignment of amino acids that comprise human and RM IgG1 hinge regions. **(B)** Strategy for generation of RM IgG1 hinge variants by repetition of 18 amino acid RM hinge domains with comparison to human IgG1 and IgG3. **(C)** RM IgG1 hinge variants were produced by plasmid transfection of 293T cells and IgG1 molecular weight was characterized by Coomassie-stained reduced SDS-PAGE. **(D, E)** Negative stain electron microscopy 2D class averages of **(D)** RM 0X and **(E)** RM 1X IgG1 hinge variants. Selected images were enlarged to show detail. **(F)** RM IgG1 hinge variants mAbs have similar binding affinities for RM Fc*γ*Rs. RM Fc*γ*R variants are identified by SNPs in the IgG contact region. Repeated symbols indicate results of technical replicates, and lines represent the means.

This functional homology for human IgG3 ADP across humans and RM is noteworthy because RM IgG3 is not structurally analogous to human IgG3. RM IgG3 lacks hinge region exon repetition, and is therefore not elongated when compared to other RM IgG subclasses (21). Therefore, to evaluate the role of antibody hinge length in determining ADP activity of RM phagocytes, we produced RM IgG1 antibodies with elongated hinge regions. To generate these reagents we used recombinant antibody production techniques to clone and express RM IgG1 with repetition of hinge region exons between the CH1 and CH2 regions. Comparison of RM and human IgG1 hinge exons are shown in **Figure 6A** and sequence alignments of the constructs used to produce RM IgG1 with elongated hinge regions are shown in **Figure 6B**. This strategy is similar to that previously used to define the contribution of hinge length to human IgG1 and IgG3 antibodies (37). We used the same nomenclature to describe these novel RM IgG1 variants —0X represents the normal RM IgG1, with no repetition of the hinge exon. 1X includes 1 additional hinge region exon, 2X includes 2 additional hinge region exons, and so on. Therefore, the 3X RM IgG1 variant is expected to be the RM analog of the most common form of human IgG3 (22, 60). Hinge variant molecular weights were characterized by Coomassie-stained reduced SDS-PAGE, and all variants were found to be of the expected size (**Figures 6C**). We used negative stain electron microscopy to visualize the hinge region elongation. 2D class averages of RM IgG1 0X mAbs are shown in **Figure 6D**, and RM IgG1 1X mAbs are shown in **Figure 6E**. A selected example of each has been enlarged so that differences in the hinge region, located between the two Fab arms and the Fc region (indicated by white arrows), can be clearly seen. Variants with additional exon repeats (2X–4X) could not be clearly resolved by negative stain EM, likely due to flexibility conferred by the longer hinges. SPR analysis demonstrated that all hinge variants had similar binding affinities for RM Fc*γ*Rs, and were similar to binding affinities of polyclonal IgG and purified IgG1 and IgG2 from RM sera (**Figure 6F**). Collectively, these data demonstrate the successful production of RM IgG1 mAbs with elongated hinge regions. We next used these novel reagents to determine if increased hinge length improves monocyte and PMN ADP activity of RM IgG1 mAbs.

### Impact of Hinge Region Length on RM Antibody Functions

**Figure 7:**
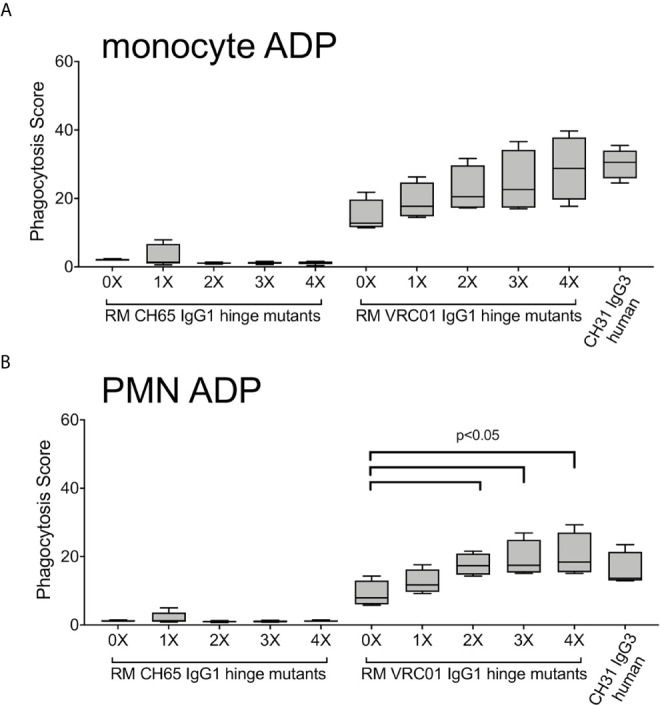
ADP activity of immune complexes formed with fluorescent HIV-1 BaL virions and RM IgG1 hinge-variant mAbs in assays performed with **(A)** monocytes and **(B)** PMN isolated from 2 RM, evaluated in two independent experiments. Box plots represent the interquartile ranges, horizontal lines indicate the medians, and error bars extend to the 10^th^ and 90^th^ percentiles.

We used our previously described HIV-1 BaL virion ADP assay to compare phagocytic functionality of RM VRC01 IgG1 hinge variants. RM IgG1 hinge variants based on the anti-influenza mAb CH65 were used as negative controls. Monocytes and PMN were isolated from peripheral blood of two healthy male RM and used as sources of phagocytes. We found that RM VRC01 IgG1 ADP activity increased concomitant with increased antibody hinge length in assays performed with RM monocytes (**Figure 7A**) and RM PMN (**Figure 7B**). Monocyte and PMN ADP activity of all VRC01 hinge variants was significantly higher than that observed for the CH65 negative controls (Wilcoxon p<0.05). Notably, we observed that the 2X, 3X, and 4X IgG variants had significantly higher RM PMN ADP activity than the 0X variant which represents the hinge length present in normal RM IgG1 and IgG3. Moreover, monocyte and PMN ADP activities of the RM IgG1 3X variant, which is most structurally analogous to human IgG3, were similar to that observed in assays performed using human CH31 IgG3. These data demonstrate that increasing antibody hinge length can be used as a strategy to improve the ADP activity of RM monoclonal antibodies.

**Figure 8:**
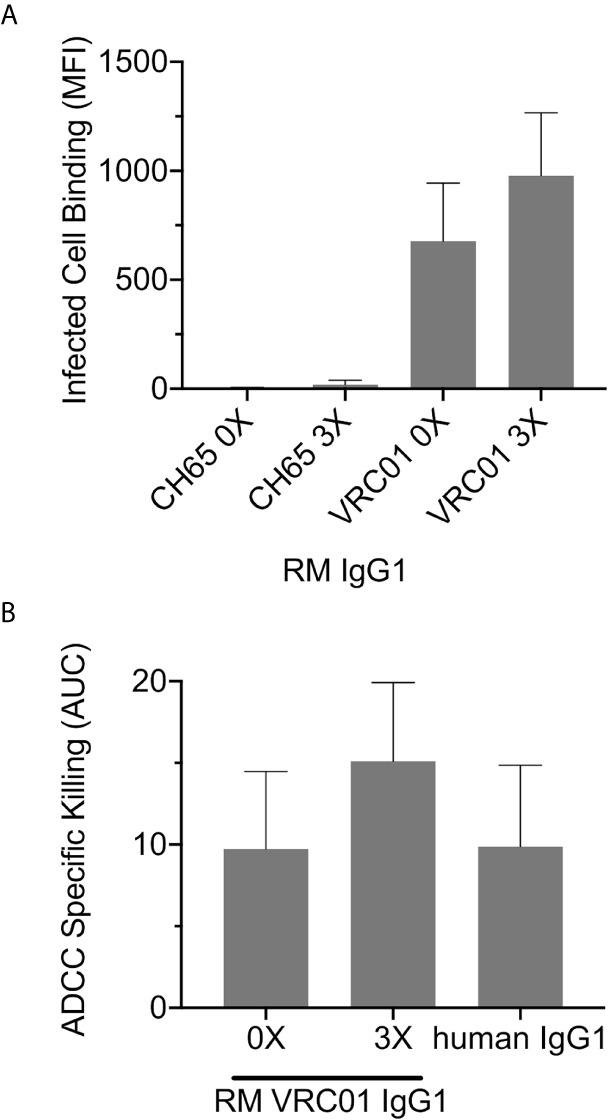
**(A)** Infected cell binding and **(B)** ADCC activity of RM IgG1 are not significantly (Wilcoxon p>0.05) impacted by elongation of the RM IgG1 hinge region. Data represents results the mean and range from two independent experiments.

We next explored whether the ability of antibody to recognize infected cells and mediate antibody-dependent cellular cytotoxicity (ADCC) was impacted by elongation of the RM IgG1 hinge region. We found no impact of increased hinge length on the ability of RM IgG1 antibody to bind to the surface of a RM CD4-expressing T cell line infected with SHIV BaL virus (**Figure 8A**), or to mediate ADCC against these target cells in the presence of RM PBMC as a source of effector cells (**Figure 8B**).

Collectively, these data suggest that RM IgG1 elongated hinge variants improve ADP activity and do not negatively impact other non-neutralizing antibody functions including infected cell recognition and ADCC. Our use of endogenous rhesus sequences to extend the hinge region is likely to mitigate the potential for immunogenicity if administered to RM *in vivo*. Therefore, these novel reagents are expected to be useful surrogates for evaluating the protective or therapeutic potential of IgG3 antibodies of clinical interest in the preclinical RM model.

## Discussion

Improved prevention strategies, including the expanded use of antiretroviral drugs, have reduced HIV-1 transmission (61), yet there remains a need for additional approaches to limit HIV-1 spread as part of an integrated program to end the global epidemic (62). Development of an effective vaccine against HIV-1 remains the foremost priority. Numerous vaccine candidates have been tested in RM models and humans, but vaccine studies performed in RMs (8, 63–67) have historically been inconsistent predictors of outcomes of human trials (68–71) —suggesting the need for a better understanding of comparative biology across these two species. Several vaccine studies performed in preclinical RM models and human clinical trials have identified associations between FcR-mediated antibody effector functions and reduced risk of infection (3–14). Among these FcR-mediated effector functions, vaccine elicited ADP activity has recently emerged as a common correlate of reduced infection risk in both species (3, 4, 6, 8, 16, 54). Of particular interest, it has recently been shown that ADP by monocytes and PMN can contribute to vaccine-mediated protection of RM (3). Ackerman and colleagues immunized RM with a DNA prime-Ad5 SIVmac239 Env-based vaccine regime *via* the intramuscular (IM) route, or intranasally in an aerosol (AE) formulation. Both regimens effectively reduced infection risk from smE660 intra-rectal challenge. ADP was identified as a correlate of this outcome in both the IM and AE vaccine groups. However, the phagocytes and antibody isotypes associated with this protective response differed by vaccine regimen. For animals vaccinated by the IM route, monocyte ADP and IgG were associated with reduced risk of infection, while ADP by neutrophils and IgA were associated with reduced risk of infection in animals vaccinated *via* the AE route. It is not yet known whether these intriguing observations would be recapitulated using similar vaccines in humans. Here, we compared ADP activity of human and RM monocytes and PMN to bridge the gap in knowledge between RM and human ADP and to provide insight into how effectively this important FcR-mediated effector response in the RM model may predict that of humans.

We measured ADP activity with *in vitro* assays that used primary monocytes and PMN isolated from fresh whole blood of 23 humans and 23 RM as sources of phagocytes. Targets in these ADP assays were fluorescent HIV-1 BaL virions that had been incubated with CD4bs specific antibodies to form immune complexes. The antibodies were produced using recombinant techniques as human IgG1, IgG3, IgA1, IgA2, and RM IgG1. Although we observed some differences in the magnitude of ADP when comparing the responses observed in assays performed with human monocytes and PMN to those in assays performed with cells from RM, we observed a consistent ranking in activity by antibody isotype and subclass. Specifically, human and RM ADP activity was greatest when mediated by human CH31 IgG3, followed by RM VRC01 IgG1 and human CH31 or VRC01 IgG1. ADP activity was lowest when mediated by human IgA mAbs. These results demonstrate functional homology between CD4bs antibodies and FcRs from humans and RM for ADP activity, irrespective of species mismatch between antibody and FcRs. This finding suggests that ADP activity observed in preclinical RM models may be translatable to activity observed in human clinical studies.

We also discovered that there was substantial variability in ADP activity when comparing between individual humans or RMs. Strong correlations across all ADP-mediating antibody isotypes and subclasses provided evidence that differences in ADP activity observed for individual humans and RM is a result of intrinsic variability in the activity of phagocytes in each blood donor and is independent of characteristics of the antibody comprising the immune complex. We therefore investigated what factors may contribute to the observed differences in ADP activity of RM and human peripheral blood phagocytes. We first explored whether FcR allelic diversity impacted ADP activity. SNPs in human FcRs result in allelic variants with lower or higher relative affinities (1, 2, 26, 72, 73). Prior studies have described associations among specific FcR alleles and infection or progression of disease resulting from diverse types of viruses (51, 53, 74–78). Importantly, genetic polymorphisms of Fc*γ*RIIa, the receptor implicated in IgG-mediated ADP of HIV-1 (52, 79), have been shown to associate with disease progression (53) and with risk of infection in the setting of vertical transmission (51). Moreover, monocyte ADP was found to be a correlate of reduced infection risk in HVTN505 Phase 2b efficacy trial, and SNPs in the Fc*γ*RIIa gene (*FCGR2A*) modified this correlation (16, 54). We therefore hypothesized that genetic variation in *FCGR2A* contributed to the differential ADP activity we observed among phagocytes from different human and RM blood donors. Instead, we found no evidence that human or RM ADP activity was impacted by *FCGR2A* genotype. We next used a quantitative flow cytometry approach to determine whether cell-surface abundance of Fc*γ*R contributed to the level of ADP activity observed in our *in vitro* assays. We found no significant correlations between levels of cell-surface Fc*γ*RII and ADP by human monocytes. Similarly, there was no consistent evidence of a relationship between RM monocyte ADP activity and Fc*γ*RII levels. In contrast, we observed that ADP activity of human IgG antibodies was positively correlated with the amount of Fc*γ*RII on the surface of human PMN and negatively correlated with cell-surface levels of Fc*γ*RI and FcαR. As we also demonstrated that the levels of Fc*γ*RII on the surface of human PMN were inversely related to the levels of Fc*γ*RI and FcαR, our findings are consistent with cell-surface levels of Fc*γ*RII having an impact on the IgG1 ADP activity of human PMN. Surprisingly, we did not find similar correlations between RM PMN Fc*γ*RII expression levels and ADP activity. Instead, we observed weak but significant correlations between the levels of Fc*γ*RI and on the surface of RM PMN and human IgG1 and IgG3 ADP activity. This result suggests that RM PMN ADP mediated by human IgG may be more dependent on signaling through Fc*γ*RI than Fc*γ*RII and warrants further exploration in future studies. Overall, our results indicate that abundance of FcR on the surface of human and RM phagocytes may contribute to a portion of the variability in ADP activity levels we observed *in vitro* for different blood donors, but it is likely that yet unknown characteristics of human and RM phagocytes are also involved. We believe that our results have important implications for design of passive or active immunization studies, especially in RM where the number of animals is typically small. RM studies intended to evaluate ADP as a correlate of protection should consider performing a screening assay to ensure that animals with low and high ADP activity are equally distributed in vaccine groups as our data demonstrated that accurate predictions of phagocyte ADP functionality cannot be made by genetic screening or by immunoprofiling phagocytes with flow cytometry.

Another principal result of our study was the demonstration that for human and RM monocytes and PMN, the highest ADP activity was consistently observed in assays performed with immune complexes formed with human IgG3. This finding is particularly relevant as accumulating evidence suggests that IgG3 responses likely contributed to reduced infection risk both in the RV144 (9, 80) and HVTN505 clinical trials (16, 54). And, although largely understudied, IgG3 is likely an important component of protective immunity against other pathogenic viruses, bacteria, and parasites (81). The superior ADP activity of human IgG3 when compared to other human IgG subclasses has been well-established in prior studies performed with human phagocyte cell lines and primary human phagocytes (41, 57, 58), and has been recently shown to be dependent on the unique elongation of the IgG3 hinge region and not due to a higher affinity for human Fc*γ*Rs (37). RM IgG3 lacks hinge region exon repetition is therefore not structurally analogous to human IgG3 (21). This fundamental difference between human and rhesus IgG3 has implications for translation of preclinical studies aimed at defining antibody-mediated protection from infection or treatment of disease performed in the RM model. Specifically, passive immunization studies aimed at exploring the antiviral functionality of IgG3 using the RM model have been limited to the use of human IgG3, which like human IgG1 is expected to be immunogenic (34). To overcome this limitation, and to determine if antibody hinge length impacts ADP activity of RM phagocytes as previously demonstrated for human IgG and human phagocytes, we produced RM IgG1 antibodies with elongated hinge regions. We cloned repeats of the RM hinge region exon between the CH1 and CH2 domains of RM IgG1 to generate these novel antibodies. This approach is similar to that used to define the contribution of hinge length to ADP activity of human IgG1 and IgG3 (37). We found that elongation of RM IgG1 improved ADP activity in assays performed with RM monocytes and PMN. Similar to that described for human IgG1 and IgG3 hinge variants, elongation of the RM IgG1 hinge region had no impact on affinity to either human or RM Fc*γ*Rs, and did not impact the ability of these antibodies to recognize Env-expressing cells and mediate ADCC. Our use of only native sequence already present in RM IgG1 is predicted to result in reduced immunogenicity in RM as previously shown for rhesus-ized IgG1 antibodies (34). Thus, we propose that these novel reagents should be used in future passive immunization studies aimed at defining the role of IgG3 and ADP in protection from virus challenge or control of disease in RM models.

The biophysical mechanism that promote improved ADP activity of antibodies with elongated hinges is not entirely clear. Elongated hinges confer flexibility to the antibody (82) that may facilitate increased access or interaction with Fc*γ*Rs at the cell surface. Alternatively, there is also evidence that the hinge may impact epitope recognition. A recent study by Richardson and colleagues demonstrated that class-switching a V2-targeting broad neutralizing antibody from IgG1 to IgG3 improved Fc dependent effector functions including ADP and ADCC, as well as neutralization potency (83), suggesting that for some epitopes the hinge region may impact paratope-epitope interactions.

There are two main limitations to our work. The first is that our experiments were performed using antibodies specific for only one Env epitope region, the CD4bs. We were faced with restrictions on the volume of blood that could be obtained from the RM used for this study and were therefore limited in the number of mAbs that could be tested. We focused on the CD4bs antibodies CH31 and VRC01 as both of these antibodies have previously been shown to have potent ADP activity (37, 41). Additionally, VRC01 was previously used to define the contribution of hinge length to human ADP and is of high clinical relevance as a candidate for passive immunotherapy against HIV-1 (37, 84, 85). While the ability of hinge extension to enhance phagocytosis has been shown for antibodies targeting the CD4bs [current study and (37)], V3 loop (37), and V2 region (83), future work will be needed to compare ADP of antibodies specific for additional epitope regions using cells from RM and humans to determine if our findings are applicable to epitopes beyond the CD4bs. The second limitation is that we only had access to peripheral blood and could therefore only compare ADP activity of human and RM circulating monocytes and PMN. Comparisons between phagocytes present in different anatomical and tissue compartments remain to be explored.

In summary, our work has several implications for translation of ADP response from studies performed in RM to humans. We demonstrated that there are major similarities between ADP activity of human and RM peripheral blood phagocytes, suggesting that RM may effectively predict responses in humans. However, our results also demonstrated substantial variability in ADP levels across individual RM and humans that could not be explained by FcR genotype or expression levels. This finding suggests that caution should be taken when assigning individuals to study groups, especially in RM studies that typically have highly restricted animal numbers as there is the potential to introduce unintentional biases. We also found that human IgG3 mediated the most robust ADP in both species. We therefore produced RM IgG1 with human IgG3-like hinge regions and demonstrated that ADP activity increased concomitant with the elongation of the hinge region. These novel reagents can be used in future passive immunization studies to investigate the protective or therapeutic potential of IgG3 antibodies of clinical interest in the preclinical RM model.

## Data Availability Statement

The raw data supporting the conclusions of this article will be made available by the authors, without undue reservation.

## Ethics Statement

The studies involving human participants were reviewed and approved by Duke Health Institutional Review Board. The patients/participants provided their written informed consent to participate in this study. The animal study was reviewed and approved by Duke University Institutional Animal Care and Use Committee.

## Author Contributions

JP conceived the study, performed experiments, analyzed and interpreted data, and wrote the manuscript. MZT, RWE, DG, AC, RJE, DE, HC, TH, CJ, EM, MT, and ED performed experiments and analyzed data. SJ and RLS performed statistical analyses. TJH developed and characterized the fluorescent HIV-1 virions. KW and MH performed next generation sequencing analyses and interpretation. KS led the design, development, and production of the rhesus-ized monoclonal antibodies. MM, MA, GF, and GT interpreted data and contributed to design of the study. All authors contributed to the article and approved the submitted version.

## Funding

This work was supported by NIH NIAID P01 grant AI120756, K01 grant OD024877, a fellowship from the Agency for Science, Technology and Research, Singapore, and the Duke University Center for AIDS Research (CFAR; NIH 5P30 AI064518).

## Conflict of Interest

The authors declare that the research was conducted in the absence of any commercial or financial relationships that could be construed as a potential conflict of interest.
