# Supplementary material for: Functional Homology for Antibody-Dependent Phagocytosis Across Humans and Rhesus Macaques
Source: Front Immunol. 2021 May 20;12:678511. doi: 10.3389/fimmu.2021.678511 (PMC8174565; doi:10.3389/fimmu.2021.678511)
Supplement: Supplementary file 1 [file DataSheet_1.pdf]

Supplemental Figure S1

>CH65VH
EVQLVQSGAEVKKPGASVKVSCKASGYTFT**DYHIN**WVRQAPGQGLEWMG**WIHPNSGDTNYAQKFQG**WVTMTRDTAISTAYMEVNGLKSDDTAVYYCAR**GGLEPRSVDYYYYGMDV**WGQGTTVTVSS
>CH65 VH RhUCA
QVQLVQSGAEVKKPGSSVKVSCKASGYTFT**DYYMH**WVRQAPRQGLEWMG**WINPYNGNTKYAQKFQG**RVTMTRDTSTSTAYMELSSLRSEDTAVYYCAR**GGLEPRSVDXYYYGLDS**WGQGVVVTVSS
>CH65 VH RhUCA_HuCDR
QVQLVQSGAEVKKPGSSVKVSCKASGYTF**TDYHIN**WVRQAPRQGLEWMG**WIHPNSGDTNYAQKFQG**RVTMTRDTSTSTAYMELSSLRSEDTAVYYCAR**GGLEPRSVDYYYYGMDV**WGQGVVVTVSS

>CH65VL
QSVLTQPPSVSVAPGQTARITC**GGNDIGRKSVH**WNQQKPGQAPVLVVC**YDSDRPS**GIPERFSGSNSGNTATLTISRVEAGDEADYYC**QVWDSSSDHVI**FGGGTKLTVL
>CH65 VL RhUCA
SYELTQPPSVSVSPGQTARITC**GGDNLGSKYVH**WYQQKPAQAPVLVIY**YDSDRPS**GIPERFSGSKSGNTATLTISGVEAGDEADYYC**QVWDSSSDHVX**FGGGTRLTVL
>CH65 VL RhUCA_HuCDR
SYELTQPPSVSVSPGQTARITC**GGNDIGRKSVH**WYQQKPAQAPVLVIY**YDSDRPS**GIPERFSGSKSGNTATLTISGVEAGDEADYYC**QVWDSSSDHVI**FGGGTRLTVL

Supplemental Figure S1. Rhesus-ization of CH65 human antibody variable regions. The human CH65 antibody amino acid sequence is provided as the top line. The rhesus macaque inferred germline gene is shown on the second line. The CDR-grafted rhesus-ized antibody variable region is shown on the bottom line. Gold indicates rhesus framework regions, red indicates macaque CDRs, black indicates human framework regions, and blue indicates human CDRs. When possible the structure of the antibody was inspected to identify the contact residues within the antibody paratope. The boxed amino acids denote known contact residues. When these amino acids were found outside the CDRs they were grafted onto the rhesus gene along with the human CDRs (green amino acids). In some cases rhesus germline amino acids could not be inferred and thus an X was placed at that position. We substituted the human residue in the rhesus-ized antibody in these instances.

Supplemental Figure S2

A.

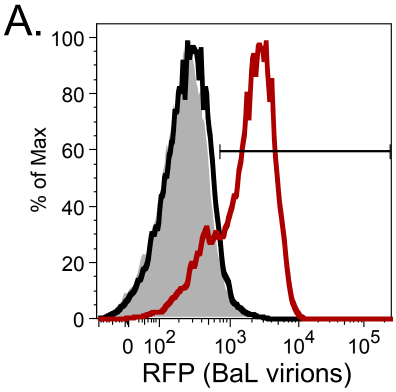

B.

Condition	% RFP Pos	MFI	% Pos x MFI	SCORE
No Antibody Control	4.8	1195	5724	NA
**HG107_IgG3** (negative control)	**4.8**	**1238**	**5992**	**1.1**
**CH31_IgG3**	**78.7**	**2694**	**212018**	**37**

Supplemental Figure S2. Example of ADP assay data. A) Histograms representing flow cytometry data for an ADP assay performed with RM PMN. The gray shaded histogram represents the no antibody control condition, the black line represents results obtained with the negative control IgG3 (HG107), and the dark red line represents results for CH31 IgG3. **B)** Values for frequency of cells positive for uptake of RFP Bal virions (% RFP Pos) and RFP MFI were used to calculate ADP score (SCORE) according to the following equation: (%Pos x MFI of antibody condition) / (%Pos x MFI of no antibody control condition).

Supplemental Figure S3

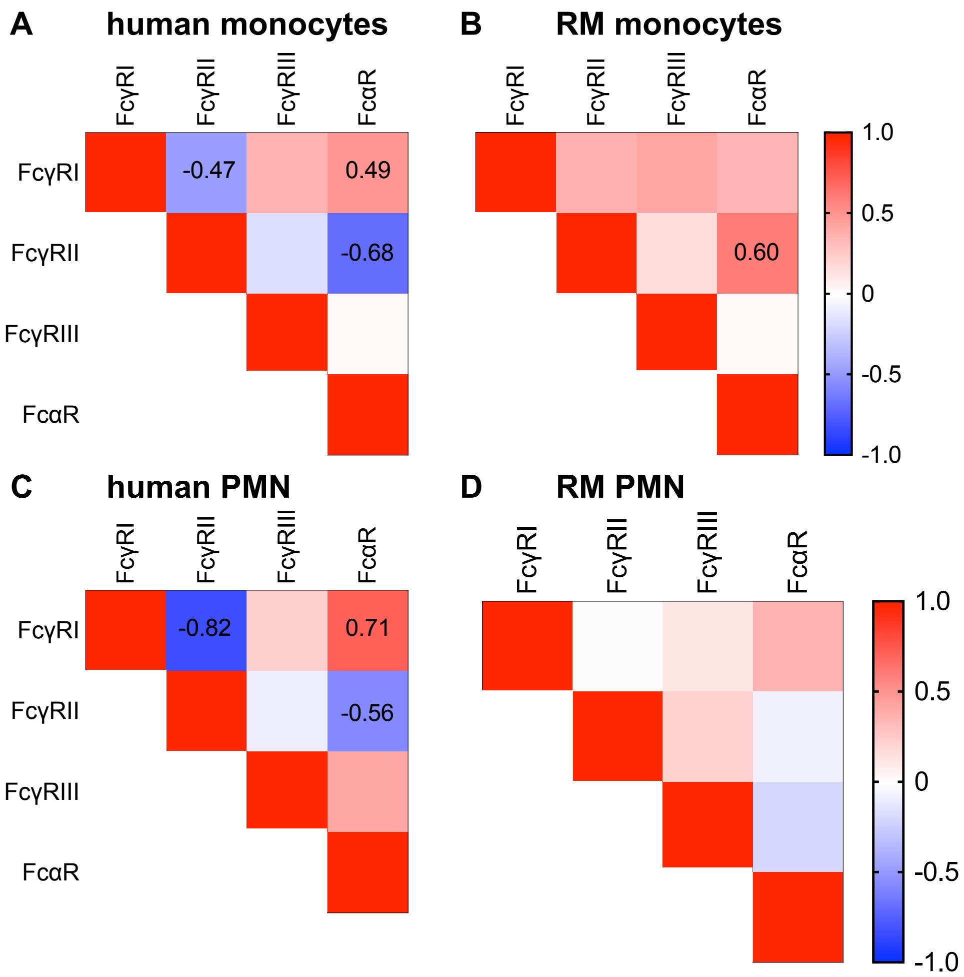

Supplemental Figure S3. Correlations between the number of cell surface FcRs on the surface of **A)** human monocytes (n=23), **B)** RM monocytes (n=21), **C)** human PMN (n=23), and **D)** RM PMN (n=22)**.** Numbers on heatmaps indicate Spearman correlation coefficients (r values) of significant correlations ($p<0.05$).

Supplemental Figure S4

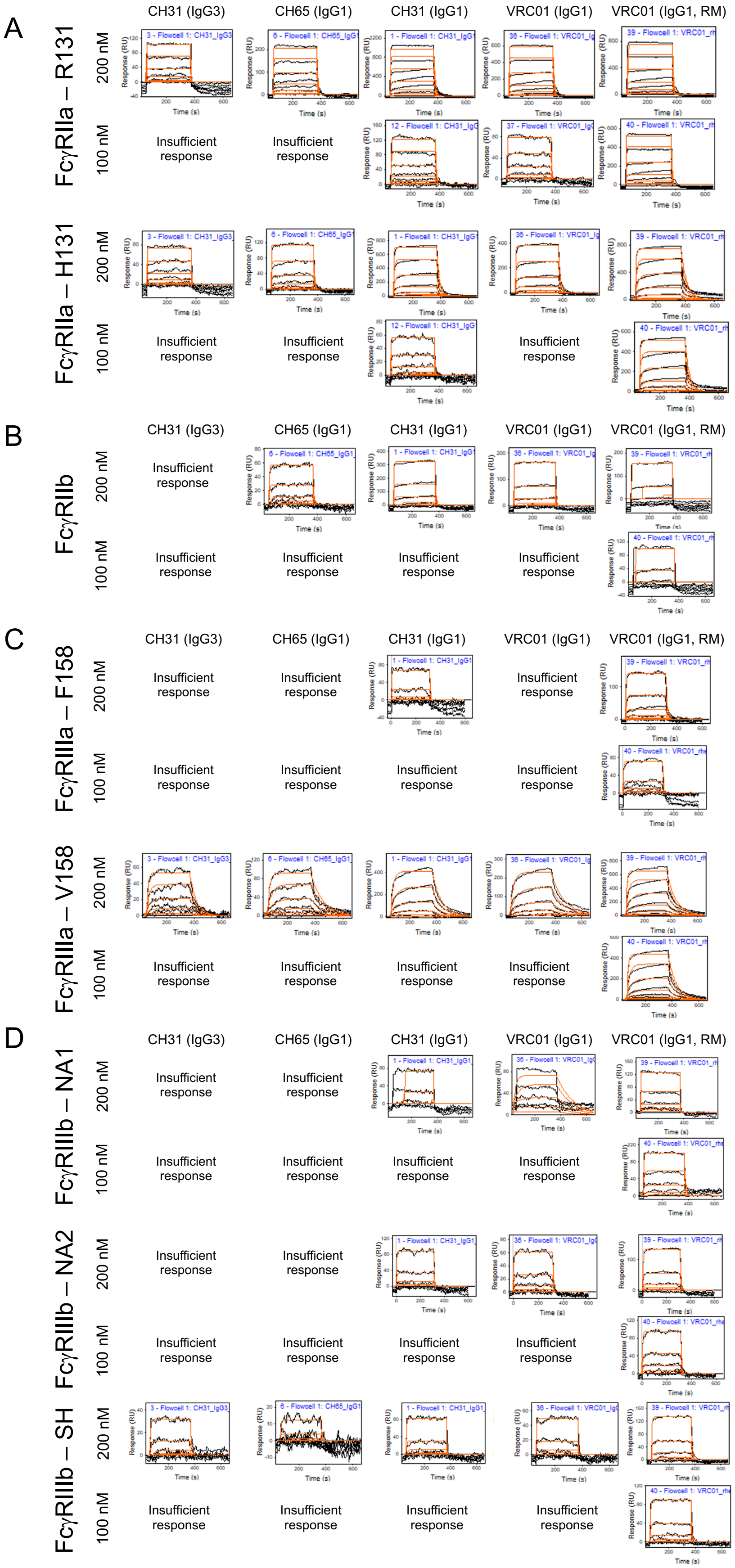

Supplemental Figure S4. Sensor data from the interaction of human **A)** FcγRIIa, **B)** FcγRIIb, **C)** FcγRIIIa, and **D)** FcγRIIIb. with each print density and the indicated antibodies. The orange curves are the kinetic fits; however, the reported values were calculated using the steady state signal at the end of the association phase. FcγRIIa and FcγRIIIa variants are identified by SNPs in the IgG contract region, and FcγRIIIb variants are identified by canonical haplotype name.

Supplemental Figure S5

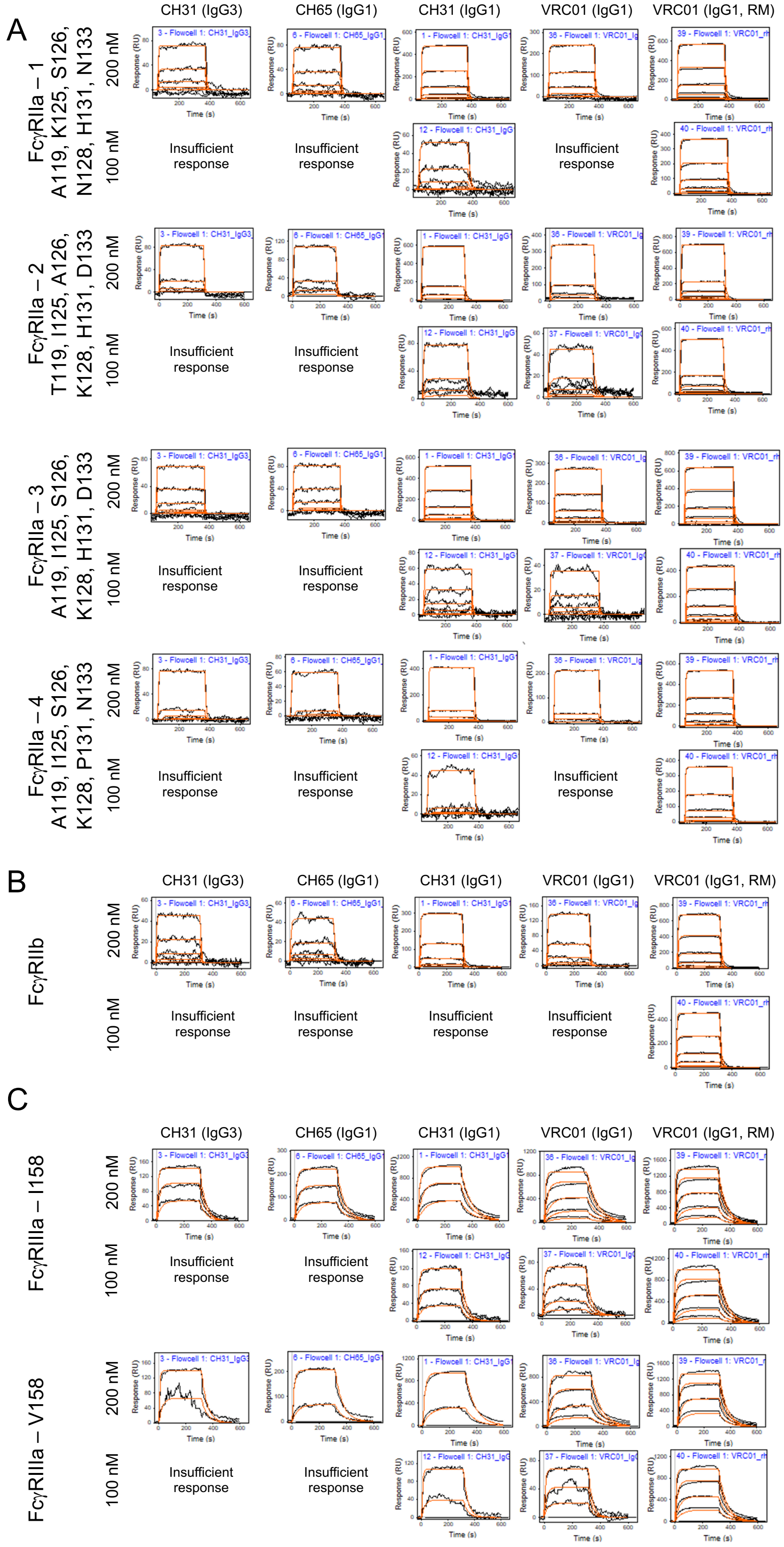

Supplemental Figure S5. Sensor data from the interaction of RM **A)** FcγRIIa, **B)** FcγRIIb, and **C)** FcγRIIIa with each print density and the indicated antibodies. The orange curves are the kinetic fits; however, the reported values were calculated using the steady state signal at the end of the association phase. FcγR variants are identified by SNPs in the IgG contact region.
